# Tissue-specific factors that govern the sialoglycan–Siglec axis in cancer

**DOI:** 10.1042/BSR20250119

**Published:** 2026-05-14

**Authors:** Jamie Wills, Adam Duxfield, Manuella Siaka Monthe, Emma Scott

**Affiliations:** Biosciences Institute, Newcastle University, Newcastle NE1 3BZ, U.K.

**Keywords:** cancer, glycobiology, immunology, sialoglycans, Siglec, tumour immunology

## Abstract

Aberrant sialylation is a persistent glycosylation change in cancer that reshapes interactions within the tumour microenvironment through the display of sialylated glycans (sialoglycans) on malignant and stromal cells. Many sialoglycans engage sialic acid-binding immunoglobulin-like lectins (Siglecs), a family of receptors expressed predominantly by immune cells that frequently transmit inhibitory signals and function as glyco-immune checkpoints. Increasing evidence indicates that tumour hypersialylation suppresses myeloid and lymphoid anti-tumour activity, promotes immune evasion, and contributes to metastatic behaviour. However, both sialoglycan repertoires and Siglec expression patterns vary markedly across cancer types and disease states, suggesting strong dependence on tissue context and tumour composition. In the present review, we discuss how tissue-of-origin programmes and lineage state establish basal sialyltransferase expression and constrain the sialoglycan landscape available to tumours. We highlight emerging single-cell evidence that stromal populations, particularly cancer-associated fibroblasts, can acquire hypersialylation and actively generate immunosuppressive Siglec ligands. We also examine how transcriptional and oncogenic regulators, including SOX2, MYC, and androgen receptor signalling, reprogramme sialyltransferase expression to produce tumour-specific sialoglycan profiles. Finally, we consider how standard-of-care therapies alter both ligand availability and immune composition, thereby dynamically modifying the sialoglycan–Siglec axis during treatment and resistance. Understanding these context-dependent determinants will be critical for interpreting sialylation in cancer biology and for designing effective therapeutic strategies targeting sialoglycan–Siglec interactions.

## Introduction

Altered glycosylation is a well-established feature of cancer and contributes to multiple aspects of tumour biology, including cell signalling, adhesion, immune recognition, and metastasis [1–4]. Among these changes, aberrant sialylation is one of the most consistently observed glycan modifications in cancer. Sialic acids are terminal monosaccharides added to glycoproteins and glycolipids through the activity of sialyltransferases, and their expression at the cell surface influences interactions between tumour cells and the surrounding microenvironment [5,6]. In particular, sialylated glycans can engage sialic acid-binding immunoglobulin-like lectins (Siglecs), a family of receptors expressed predominantly by immune cells, many of which transmit inhibitory signals that dampen immune activation [7].

Increasing evidence indicates that the sialoglycan–Siglec axis plays an important role in shaping anti-tumour immune responses. Tumour-associated hypersialylation has been shown to impair myeloid and lymphoid cell function through Siglec-mediated signalling, contributing to immune suppression within the tumour microenvironment [8–14]. However, the extent and nature of sialoglycan expression, as well as the Siglec repertoire present within tumours, vary considerably between cancer types. These differences suggest that tissue-specific factors strongly influence the regulation and function of this pathway.

Basal glycosylation programmes differ between tissues, reflecting developmental origin, cellular differentiation state, and lineage-specific expression of glycosylation enzymes. The expression levels and substrate specificities of sialyltransferases, together with the availability of acceptor glycans, determine the composition of sialoglycans present on the cell surface [15]. In cancer, these pre-existing glycosylation patterns may be modified by oncogenic signalling, epigenetic changes, and microenvironmental cues, resulting in tumour-specific sialoglycan profiles that retain features of their tissue of origin.

Cellular heterogeneity within tumours further adds to the complexity of sialoglycan expression. Distinct tumour cell subpopulations, stromal cells (including fibroblast subsets), and infiltrating immune cells can display different sialylation patterns, influencing local immune interactions [16,17]. Importantly, transcription factors and lineage-determining regulators that control cellular identity have been shown to influence the expression of glycosylation-related genes, linking differentiation state directly to sialoglycan composition in cancer cells.

The functional impact of tumour-associated sialoglycans also depends on the immune landscape of the tumour microenvironment. Siglec expression varies across immune cell types and is dynamically regulated by tissue context, inflammatory signals, and disease state [18]. As a result, the availability of Siglec receptors capable of recognising tumour-associated sialoglycans differs between tumour types and tissue sites, affecting the degree to which sialylation contributes to immune modulation.

In the present review, we discuss how tissue-specific factors regulate the sialoglycan–Siglec axis in cancer. We examine the cell-specific nature of sialoglycan expression in tumours, the role of regulatory programmes in shaping glycosylation profiles, the influence of the tumour immune landscape on Siglec expression, and the impact of standard-of-care therapies on this axis. Understanding how these factors intersect will be important for interpreting the role of sialylation in cancer biology and for informing future therapeutic strategies.

### Tissue-specific basal levels of sialyltransferases control sialoglycans synthesis

The composition of cell surface sialoglycans is largely determined by the basal expression of sialyltransferases, a family of Golgi-resident enzymes that catalyse the transfer of sialic acid from CMP-sialic acid to glycan acceptors [15]. In mammalian tissues, distinct sialylation patterns are evident under homeostatic conditions, reflecting stable, tissue-specific expression of sialyltransferase genes rather than transient regulatory inputs. These basal glycosylation programmes establish the sialoglycan landscape upon which oncogenic and microenvironmental influences act during malignant transformation.

Mammalian sialyltransferases are divided into four major families based on the glycosidic linkage formed and the nature of the acceptor substrate: ST3GAL (α2-3-linkage to galactose), ST6GAL (α2-6-linkage to galactose), ST6GALNAC (α2-6-linkage to GalNAc), and ST8SIA (α2-8-linkage to sialic acid) [5,19,20]. Members of these families exhibit distinct and often non-overlapping tissue expression patterns. For example, ST6GAL1 is broadly expressed across epithelial and hematopoietic tissues, whereas ST6GAL2 shows more restricted expression in the central nervous system [21,22]. Similarly, specific ST3GAL isoforms are preferentially expressed in gastrointestinal, respiratory, or immune tissues, contributing to regionally distinct sialoglycan repertoires [20].

**Figure 1:**
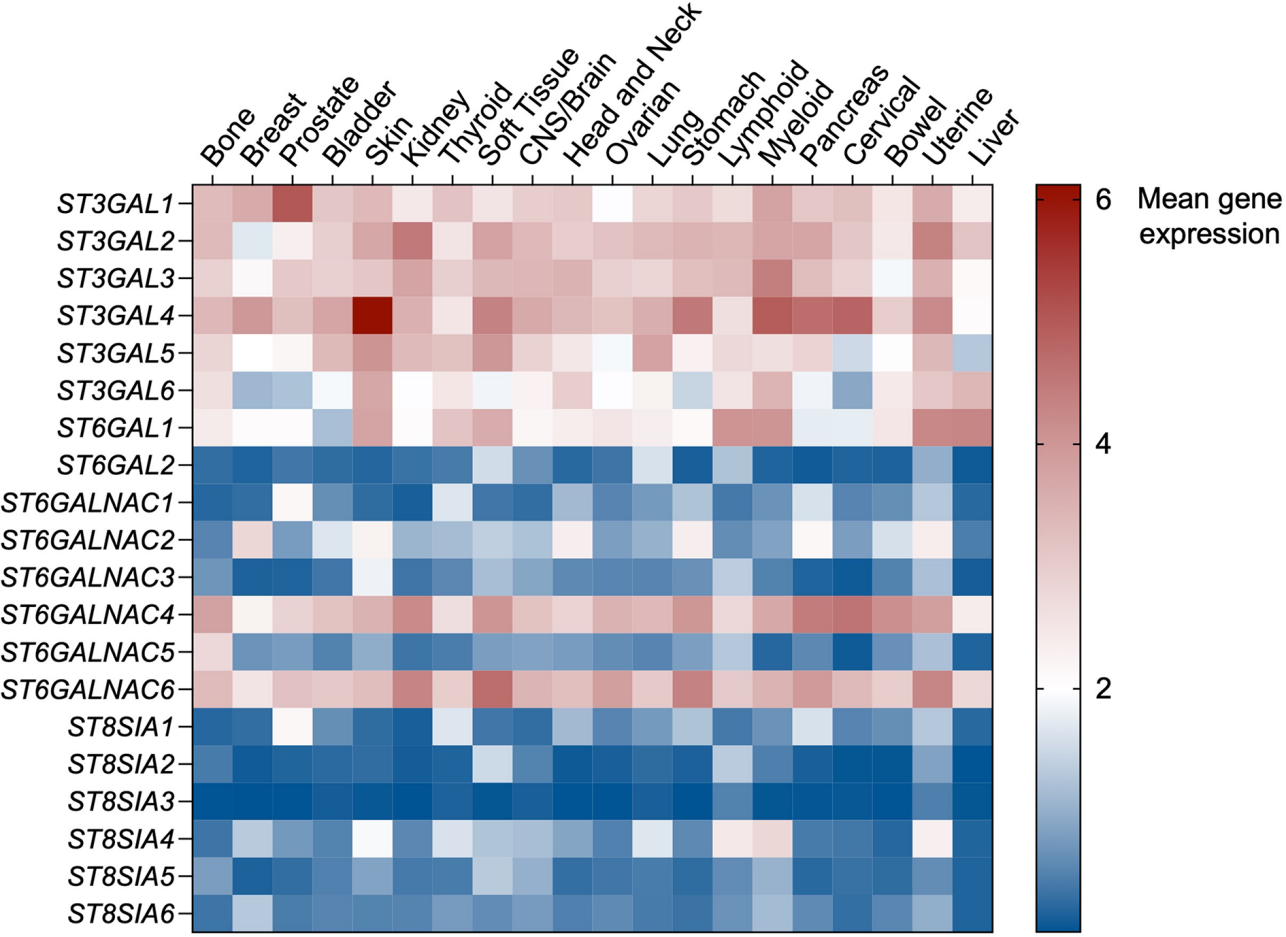
Gene expression of sialyltransferases across solid and haematological malignancies. Mean gene expression values for all 20 sialyltransferases across a range of solid and haematopoietic malignancies. Data are mean log_2_ transcripts per million +1 accessed using the PanCancer Transcriptome Atlas (https://pcatools.shinyapps.io/PCTA_app/).

Basal glycosylation programmes provide a framework that can influence the repertoire of sialoglycans available to tumours; however, malignant transformation frequently involves transcriptional, epigenetic, and microenvironment-driven reprogramming of sialyltransferase expression, meaning that tumour sialylation does not always mirror that of the corresponding normal tissue. Notably, several cancers can exhibit marked hypersialylation despite relatively low expression of specific sialyltransferases in the healthy tissue of origin, highlighting that lineage constraints can be overridden during tumour progression. This is highlighted in Figure 1, which shows mean gene expression of all 20 sialyltransferases across a range of solid and haematopoietic malignancies (Figure 1).

The diversity of mammalian sialyltransferases reflects a history of gene duplication and divergence during vertebrate evolution. Phylogenetic analyses indicate that early expansion of the sialyltransferase gene families enabled increasing complexity in sialylation patterns, supporting the emergence of specialised tissues and immune regulatory mechanisms [21,23]. While the core catalytic domains of these enzymes are conserved, variations in regulatory regions, substrate-binding sites, and Golgi localisation signals have driven functional diversification [24].

This evolutionary expansion has resulted in sialyltransferases with overlapping but non-identical substrate specificities. Closely related enzymes may catalyse similar linkages but differ in their preference for *N*-glycans, *O*-glycans, or glycolipids, as well as in their efficiency towards specific glycan scaffolds. Consequently, the phylogenetic relationship between sialyltransferases provides insight into both their potential redundancy and their capacity to generate distinct sialoglycan structures in a tissue-dependent manner.

Functional redundancy is a prominent feature of the sialyltransferase families. Multiple enzymes can catalyse the formation of the same linkage type, raising the possibility of compensatory mechanisms when individual sialyltransferases are absent or down-regulated [23,25]. This redundancy is supported by genetic studies in which deletion of single sialyltransferase genes often results in partial, rather than complete loss of specific sialylated structures. In many cases, the remaining family members are sufficient to preserve baseline sialylation, albeit with altered glycan fine structure or reduced abundance.

However, redundancy is not absolute. Differences in enzyme expression level, subcellular localisation within the Golgi, and acceptor substrate availability impose functional constraints on compensation. For example, an enzyme expressed at low basal levels in a given tissue may be unable to fully substitute for a highly expressed isoform, even if catalytic capabilities overlap. Furthermore, some sialyltransferases exhibit unique roles in generating specialised glycans, such as disialylated or polysialylated structures, that cannot be readily replaced by other family members [25,26].

The combination of tissue-specific basal expression, phylogenetic diversification, and partial redundancy among sialyltransferases creates a constrained yet flexible system for sialoglycan synthesis. In cancer, alterations in sialylation often reflect amplification or redistribution of these pre-existing programmes rather than *de novo* glycosylation pathways. As such, the sialoglycan profiles observed in tumours are shaped not only by oncogenic signals but also by the inherited sialyltransferase expression patterns of the tissue of origin. Understanding these basal and evolutionary determinants of sialylation is essential for interpreting tumour-associated glycan changes and for predicting how different cancers may engage immune receptors such as Siglecs. It also has implications for therapeutic strategies aimed at targeting sialylation, as the degree of redundancy and tissue specificity within the sialyltransferase families may influence both efficacy and selectivity of such interventions.

### Cell-specific expression of sialyltransferases in the tumour immune microenvironment

With the advent of single-cell sequencing technologies, several studies have revealed that sialyltransferases and accompanying sialoglycans are differentially expressed in immune evasive epithelial cancer cells as well as tumour stroma populations such as cancer-associated fibroblasts (CAFs) [27,28]. CAFs promote tumour growth through various mechanisms. They are also a major stromal component within solid tumours, remodel the extracellular matrix and increase tissue stiffness, and physically hinder drug delivery [29,30]. Despite their critical role in shaping tumour behaviour, CAFs remain understudied as contributors to tumour-associated glycosylation. Recent studies have demonstrated that CAFs undergo tumour-induced hypersialylation and differentially express sialyltransferases that generate immunosuppressive sialoglycans [16,17,31]. Seminal work from Egan et al*.* demonstrated that α2-3 and α2-6 sialic acids are up-regulated on the cell surface of CAFs compared with normal fibroblasts, accompanied by increased transcription of sialyltransferases *ST6GAL1* and *ST6GAL2*. These sialylated fibroblasts suppressed CD8^+^ T-cell proliferation and effector function in co-culture assays. Importantly, enzymatic desialylation of CAFs or pharmacological disruption of Siglec–sialoglycan interactions restored T-cell activity [17]. Building on these findings, O’Neill et al*.* identified that ST6GalNAc6 is enriched in tumour stromal cells compared with epithelial cells and that associated hypersialylation drives engagement with Siglec-10 in myeloid populations. Using *in vivo* models, stromal Siglec-ligand enrichment correlates with impaired anti-tumour immunity and reduced responses to immune checkpoint inhibition. Targeting stromal sialylation enhanced anti-tumour immune activity and improved immunotherapy response by reducing PD-L1 in macrophages and increasing granzyme B in NK cells, providing evidence that CAF-derived sialoglycans contribute to the formation of an immunosuppressive stromal niche.

Given these findings alongside the proposed heterogeneity of CAF populations, it is highly plausible that sialyltransferase expression is differentially distributed across CAF subsets and is an area currently under investigation [32]. Taken together, these studies highlight that the tumour stroma actively contributes to the formation of an immunosuppressive, sialylated tumour microenvironment and should be considered in pre-clinical evaluations of novel therapies targeting this axis.

### Lineage specific transcriptional networks determine sialoglycan profiles in cancer

The expression of specific sialyltransferases in cancer has been shown to be regulated by multiple mechanisms including transcriptional regulation, gene amplifications, microRNA’s, and epigenetic modifications [33–38]. Importantly, key lineage-determining transcription factors play a central role in controlling sialoglycan expression in cancer [39]. Growing evidence suggests that these transcriptional networks drive cancer progression in part by regulating sialyltransferases and consequently, tumour cell surface sialylation. One clear example is the stem/progenitor lineage factor SOX2. SOX2 protein levels correlate with expression of ST6Gal1 (ST6Gal-I) across ovarian cancer cell lines including Pa-1, Skov3, and OVACAR3 [40]. Dorsett et al*.* further demonstrated, using chromatin immunoprecipitation, that SOX2 binds directly to the P3 promoter of ST6Gal1, promoting its transcription [40]. Functionally, this interaction increases cell surface α2-6-linked sialic acids, showing how a lineage factor that maintains progenitor-like states can directly shape sialoglycan profiles during ovarian cancer progression.

Another prominent regulator is the oncogenic transcription factor c-MYC, which has been repeatedly linked to altered tumour glycosylation. In the 21H small cell lung cancer model, c-MYC binds directly to the promoter regions of *ST3GAL5* and *ST6GALNAC5*, and knockdown or overexpression of MYC causes corresponding changes in both enzymes and their downstream α2-3- and α2-6-sialylated structures on the cell surface [41]. This provides a mechanism through which MYC promotes hypersialylation, facilitating immune evasion via the sialic acid-Siglec-7/9 axis.

c-MYC has also been implicated in regulating sialyl Lewis X (sLeX), a metastasis-associated glycan and E-selectin ligand. Following EGF stimulation of colon cancer cell lines HT29 and DLD-1, c-MYC shows strong promoter binding at *ST3GAL1, ST3GAL3*, and *ST3GAL4*, and shRNA depletion of MYC reduces expression of these enzymes together with sLeX abundance [42]. This links MYC-driven transcriptional programmes to sLeX-mediated processes, including epithelial–mesenchymal transition and metastatic trafficking.

More recently, MYC has also been shown to regulate sialoglycans in T-cell acute lymphoblastic leukaemia. High MYC activity drives expression of ST6GalNac4, which supports surface expression of the disialyl-T glycan, a ligand with glyco-immune checkpoint function. Modulation of MYC (including doxycycline-controlled MYC systems) alters *St6galnac4* transcription and corresponding disialyl-T expression, with loss of ligands for immune inhibitory receptors such as Siglec-7 and Siglec-E [43]. These findings identify a MYC-ST6GalNac4 axis that promotes immune escape through suppression of macrophage activity.

In our recent work, we further demonstrate an important role for MYC in regulating ST3Gal1 and α2-3 sialylation in prostate cancer [44]. Prostate cancer progression is strongly influenced by androgen receptor (AR) signalling, and previous work suggests ST3Gal1 is androgen-regulated, with anti-androgen therapy increasing ST3Gal1 and α2-3 sialylation [45]. However, the contribution of AR–MYC cross-talk to sialoglycan regulation has remained poorly defined. In line with previous reports, we also confirmed that ST6GalNAc1 is a target for the AR. However, in contrast with prior reports, we found no evidence supporting androgen regulation of ST6Gal1 under physiological androgen conditions or following anti-androgen therapy [46–49].

Notably, binding site analyses indicate that *ST3GAL1* has regulatory input from both AR and MYC and functionally, this cooperative signalling increases Siglec-7 ligand abundance in an ST3Gal1-dependent manner, supporting immune evasion and prostate cancer progression [44].

These studies highlight a context-dependent, cancer-specific relationship in which transcription factors and lineage regulators control sialyltransferase expression and downstream sialoglycan presentation. This transcriptional regulation has clear functional consequences, shaping immune interactions and tumour-promoting processes such as immune checkpoint engagement, EMT, and metastatic dissemination.

### The tumour immune landscape dictates Siglec expression in tumours

**Figure 2:**
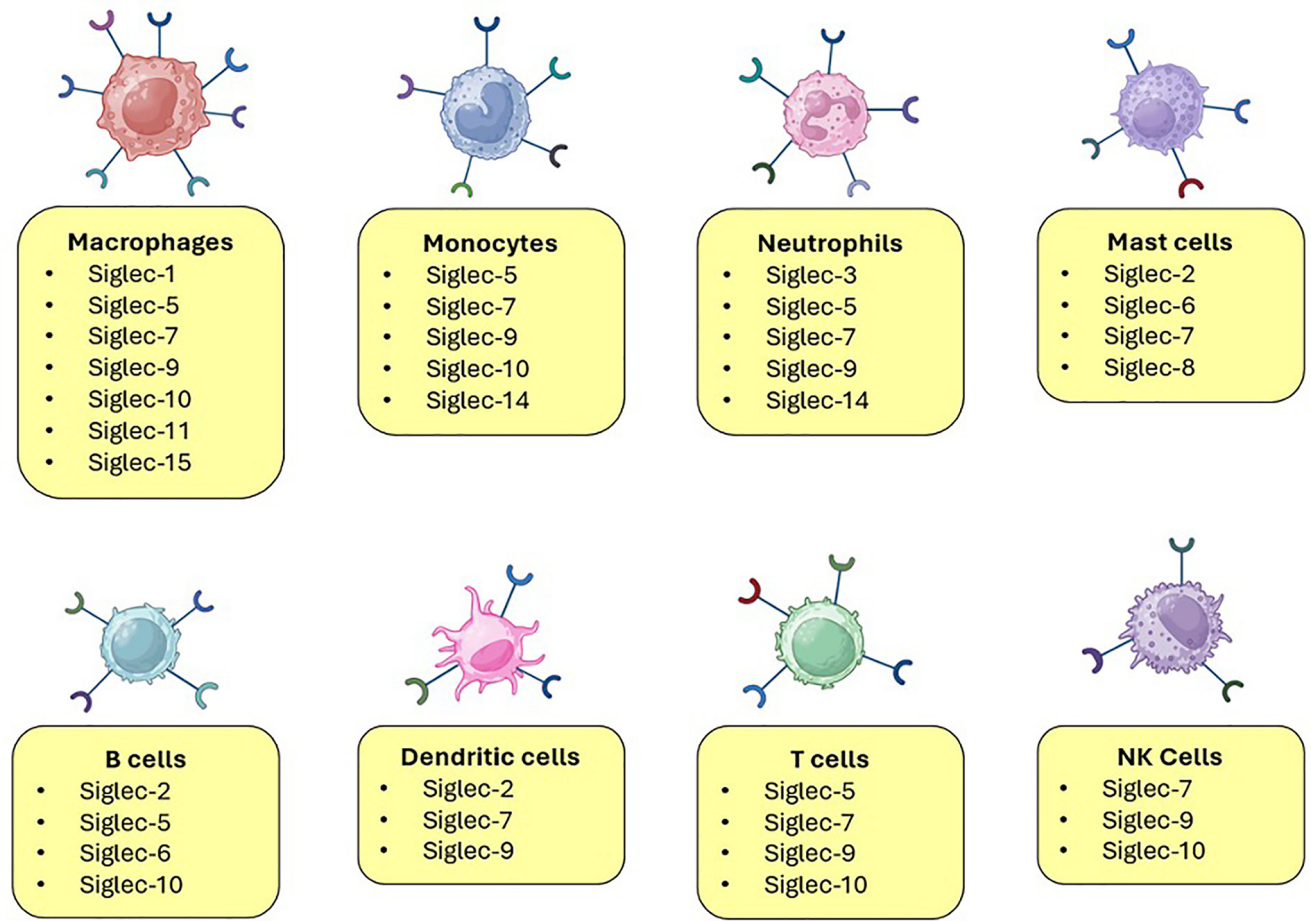
Siglec receptor expression across immune populations. Reported Siglec receptor expression across immune cell subsets.

In humans, they are mainly found on the surface of immune cells, although the expression of some Siglecs has also been reported on the surface of cancer cells [18,50,51]. In total, there are 15 human Siglecs, which display differing expression patterns across different immune populations. Beyond this, phenotypic subsets of immune populations, such as those polarised towards different states or phenotypes, can also exhibit different Siglec expression profiles (Figure 2). Most Siglecs contain an intracellular ITIM or ITIM-like motif, allowing them to function as anti-inflammatory modulators of the immune response. Siglec-14, Siglec-15, and Siglec-16 do not contain an internal ITIM or ITIM-like motif and instead interact directly with DAP proteins that contain ITAM motifs, allowing them to function as activating Siglecs [52,53].

When engaged by their sialylated ligands, Siglecs elicit an anti-inflammatory signalling response to dampen down immune activation [54]. As a result, higher Siglec expression is usually associated with subsets of immune cells involved in anti-inflammatory signalling, with pro-inflammatory immune subsets conversely associated with lower Siglec expression.

**Figure 3:**
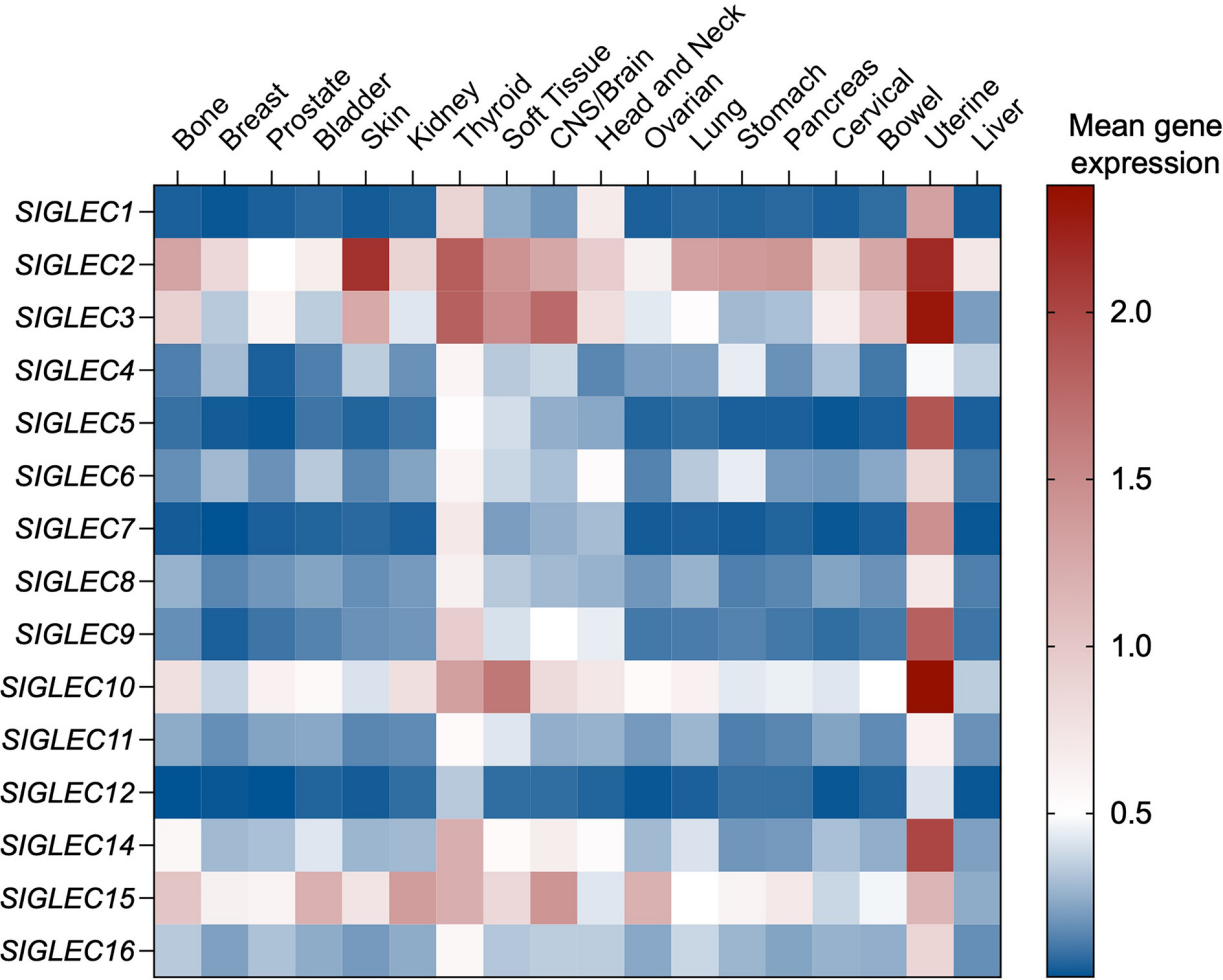
Siglec receptor gene expression across solid malignancies. Mean gene expression values for all 15 Siglec receptors across a range of solid malignancies. Data are mean log_2_ transcripts per million +1 accessed using the PanCancer Transcriptome Atlas (https://pcatools.shinyapps.io/PCTA_app/).

As the expression of Siglec receptors is mainly constrained to cells of the immune system, the tumour immune landscape strongly determines which Siglecs are found within a tumour, how abundant they are, and their contribution to tumour immune modulation (Figure 3). There are a myriad of factors within the tumour that can influence the immune landscape, determining which immune cells are recruited to the site of a tumour and how they function once there, which in turn influences which Siglecs they will express. For example, the TME of cancers associated with high levels of macrophage infiltration will express increased levels of macrophage-associated Siglecs, such as Siglec-15, while other cancers, such as those associated with elevated B-cell levels, will express higher levels of B-cell associated Siglecs, such as Siglec-2 (also known as CD22).

One factor that determines the immune makeup of a tumour is the stage of the cancer, whether early or advanced. As some cancers progress, the type and characteristics of immune cells found within their TME often change [55,56]. Early-stage cancers can contain higher levels of pro-inflammatory, immune effector cells, which can recognise and target cancer cells [57–59]. As most Siglecs are involved in anti-inflammatory signalling, Siglec expression on immune cells within these early tumours may typically be low. The expression of some pro-inflammatory Siglecs may be seen in these early tumours, such as the IFN-inducible Siglec-1, which acts as a marker of activated macrophages and is enriched in earlier, more inflammatory tumours [60,61]. However, as a cancer becomes more advanced, these immune effector cells can become suppressed or are replaced within the tumour by different, more suppressive immune cells [57–59]. These suppressive immune cells can secrete pro-tumourigenic factors and further suppress other immune cells in the TME, thereby allowing cancerous cells to escape being targeted and killed by the immune system. As most Siglec family members are associated with an anti-inflammatory response, Siglec expression in immune cells in later-stage cancers will typically be higher, allowing them to continue suppressing anti-tumour immunity within the TME. Thereby, the immune landscape of a tumour at different stages of disease will contain a different range of Siglec receptors. Siglec expression can also vary vastly between different subtypes or phenotypes of the same cell. For example, an M1-like macrophage will generally express low levels of immunosuppressive Siglecs, allowing it to retain a pro-inflammatory phenotype. However, macrophages polarised towards an anti-inflammatory M2-like state, such as those usually found within a tumour, will express significantly higher levels of many Siglecs [62,63]. This helps these cells to maintain their immunosuppressive phenotype. Suppressive immune cells within a tumour are typically pro-tumourigenic, and can suppress the activity of nearby effector cells, such as CD8+ T cells [51,64–66]. The polarisation of immune cells to different states is often driven by the secretion of different factors and cytokines within the TME, either from cancer cells or other immune cells. For example, factors such as TNF-α and IFN-γ drive an M1-like macrophage phenotype associated with low Siglec expression, while secretion of factors such as IL-4 and IL-13 promotes M2-like polarisation [67], resulting in increased expression of Siglec receptors. Importantly, this shows how factors secreted within the TME can influence the type of Siglecs present, which is largely determined by the types of immune cells present, and which state or phenotype that they exhibit.

### Standard-of-care therapies shape sialoglycan and Siglec expression in cancer

Many common cancer treatments can have an impact on the expression of Siglecs and their sialoglycan ligands. One treatment that has become a standard-of-care therapy for many cancers, and can also affect the expression of many sialoglycans, is anti-hormonal therapy. Numerous cancers depend on hormonal signalling to continue proliferating, manipulating a pathway necessary for the growth and survival of healthy non-cancerous cells. Among these are prostate cancer, driven by androgen signalling [68], and breast cancer, driven by the hormones oestrogen and progesterone [69]. As a result, many of the standard therapies for these cancers are focused on blockade of these hormone signalling pathways. In prostate cancer, androgen deprivation therapy and AR signalling inhibitors are routinely used for patients with locally advanced or metastatic disease. In breast cancer, the oestrogen receptor modulator tamoxifen is routinely offered to patients, as well as other treatments aimed at lowering oestrogen production, such as aromatase inhibitors or GnRH agonists [70]. While these treatments effectively suppress their target hormone signalling pathways initially and can slow down disease progression, they can also affect the expression of sialyltransferases and Siglecs in the TME.

The expression of many sialyltransferases are regulated by hormonal signalling pathways, meaning that targeting these pathways will also affect the expression of sialoglycans. Some sialyltransferases are positively hormone-regulated, such as ST6GalNAc1 [48]. Subsequently, expression of ST6GalNAc1-synthesised sialoglycans such as sialyl-Tn will be elevated in hormone-dependent primary tumours but will become down-regulated when the AR signalling pathway is suppressed. Conversely, ST3Gal1 is negatively regulated by androgen signalling in prostate cancer, meaning that while ST3Gal1 expression may be relatively low in untreated hormone-driven tumours, expression of sialoglycans synthesised by ST3Gal1 may become up-regulated in response to anti-hormone therapies [44,45]. It is important that the effect of hormone signalling inhibition on sialoglycans is fully understood, as many hormone-regulated sialoglycans serve as ligands for immunosuppressive Siglec receptors. If the expression of some sialoglycans is modulated by hormone blockade, such as Siglec-7 and Siglec-9 ligands, this may have implications for anti-tumour immunity, and subsequent therapeutic interventions.

While many patients initially respond well to hormone inhibition as a cancer therapy, resistance regularly arises, meaning cancer cells are no longer sensitive to hormone inhibition. Usually, this occurs due to alternative splicing of hormone receptor genes, resulting in a final protein that is altered or truncated, known as splice variants. These variants often lack a ligand-binding domain but retain their intracellular signalling domain, allowing them to be constitutively active regardless of whether their hormone ligands are present [71,72]. Accordingly, while hormone pathway inhibition may initially reduce the expression of hormone-regulated sialoglycans and sialyltransferases, their expression will eventually be restored in patients who have become resistant to this treatment. To effectively target the sialoglycan–Siglec axis in these cases, it is important that alternative therapeutic strategies are developed, as hormone inhibition does not remain effective long term.

Inhibition of aberrant signalling pathways has also become a mainstay of treatment for many cancers. Multiple signalling pathways are hyperactivated in cancer, particularly those associated with increased growth and survival, and can drive cancer progression and treatment resistance. Protein kinases and GTPases play a key role in the transduction of a signal through intracellular signalling pathways by activating downstream proteins in the pathway. Protein kinase inhibitors are used in a broad range of cancers and work by blocking the activity of a protein kinase, thereby preventing it from phosphorylating its downstream proteins. While most GTPases have historically been seen as ‘undruggable’ due to their importance in healthy cell signalling, recently, some GTPase inhibitors have been developed, which are typically targeted against their mutant cancer-associated isoforms. These agents suppress signalling via the targeted pathway and can be effective at slowing or stopping cancer progression, at least initially.

Many of the signalling pathways that are often hyperactivated in cancer also regulate the expression of sialyltransferases, meaning that therapeutically targeting these pathways can affect sialoglycan expression. For example, expression of the sialyltransferase ST6Gal1, responsible for α2-6 sialyation of many glycans, is reported to be regulated by members of the Ras family of GTPases [73]. Hyperactivation of pathways involving Ras family members is implicated in several cancers, such as non-small cell lung cancer (NSCLC) and pancreatic cancer [74,75]. This hyperactivation often occurs through mutation of Ras family members, causing them to remain in an active, GTP-bound state, in which they are able to constantly activate their downstream proteins [76]. Recently, two KRAS inhibitors have been clinically approved for the treatment of NSCLC, Sotorasib and Adagrasib. These drugs target the KRAS G12C mutant, locking it in an inactive GDP-bound state and preventing it from activating downstream proteins. Suppression of KRAS with these treatments will lead to a reduction in the expression of its downstream gene targets, including ST6Gal1, subsequently reducing the expression of α2-6-linked sialoglycans.

The expression of many Siglecs can also be influenced by the targeting of protein kinases and signalling pathways in cancer. While Siglecs are mainly expressed on immune cells rather than cancer cells, with a few exceptions, off-target effects of targeting cancer cells can expose immune cells within a tumour to these therapeutic agents. The expression of many cytokines and factors involved in immune recruitment and polarisation falls under the control of pathways that are commonly hyperactivated in cancer. For example, the pro-inflammatory cytokine TNF-α falls under the control of the NF-κB pathway, while the immunosuppressive cytokine IL-10 is regulated by ERK and p38, although these lists are not exhaustive, and many different pathways coordinate to induce the expression of cytokines. Therapeutic targeting of these pathways can thereby affect the release of these cytokines, and therefore impact the recruitment, state, and level of immune cells within a tumour, which directly dictates Siglec expression. This may hold some therapeutic benefit, as suppression of a signalling pathway that regulates anti-inflammatory cytokines, such as IL-10 or IL-13, may increase anti-tumour immunity within these tumours by reducing the expression of immunosuppressive Siglecs.

Chemotherapy is used as a therapeutic strategy in a broad range of cancers and involves the delivery of cytotoxic agents to selectively kill cancerous cells. Most forms of chemotherapy are associated with significant side effects due to off-target effects, which can also impact the expression of many sialoglycans and Siglecs. Most chemotherapeutic drugs activate stress-related pathways in cancer cells, up-regulating proteins such as NF-κB, Hif-1α, and TGF-β [77–79]. This stress-induced shift in cancer cells can lead to an increase in the levels of many sialyltransferases, and subsequently the sialoglycans they synthesise. This increase in sialyltransferase is not only induced by stress, but can also confer resistance to chemotherapy, with elevated levels of key sialyltransferases such as ST6Gal1, ST3Gal2, and ST6GalNAc1 seen in surviving cells following chemotherapy treatment [80,81]. It is hypothesised that an increase in sialoglycan levels on the surface of cancer cells can act as a physical barrier against chemotherapeutic agents, preventing them from reaching the cell and thus protecting the cell from stress-induced cell death. The thickened glycocalyx can slow the time it takes to diffuse towards the cell surface and physically trap drugs. In breast cancer cells, an increase in cell-surface sialyation blocked the chemotherapy drug doxorubicin from reaching the cell membrane and correlated with reduced cytotoxic cell death [82,83]. The heavily sialylated glycoprotein MUC1 has also been shown to reduce the accessibility of cancer cells to several chemotherapeutic agents, leading to increased survival of these cells [84]. Additionally, drugs can also remain trapped in the glycocalyx due to electrostatic attraction; while sialic acids carry a negative charge at physiological pH, many chemotherapy drugs will be positively charged. This attraction will further increase the time it takes for chemotherapeutic agents to diffuse through the glycocalyx to the cell membrane or prevent them from reaching it entirely. In this way, increased expression of sialoglycans on the cell surface increases chemoresistance and lends a selective advantage to these highly glycosylated cells. Cells with a thickened glycocalyx are more likely to survive each round of chemotherapy, causing a shift in the surviving population of cancer cells to expressing increased levels of sialoglycans.

Chemotherapy can also influence the expression of Siglecs in a tumour. The release of stress-related factors and cytokines can alter the recruitment and subsequent inflammatory state of many immune cells, which directly impacts their Siglec expression. For example, TNF-α released following chemotherapy will push macrophages towards an M1-like, pro-inflammatory state, while the release of TGF-β and IL-10 drives an immunosuppressive, M2-like state; thus, differing levels of these two factors released following chemotherapy would vastly alter the Siglec expression of macrophages in a tumour [85,86]. Different groups report conflicting evidence on the effect of chemotherapy on some immune populations, which may vary with different drugs. For example, while it is reported that chemotherapy with agents such as gemcitabine can reduce the population of immunosuppressive myeloid-derived suppressor cells (MDSCs) and T-regulatory cells within a tumour [87,88], other studies report that chemotherapy using 5-fluorouracil can induce the recruitment of MDSCs into a tumour [89]. The TME will express an entirely different range of Siglecs depending on whether these suppressive cells are being recruited to the tumour, or depleted. This underscores the highly complex effect of cytotoxic therapeutic agents on the immune landscape of a tumour; there is no ‘one-size-fits-all’ approach, with vastly different effects seen between different treatments and in different cancer settings. It is important that the effect of a particular treatment on the immune landscape of a particular cancer is known, to identify any potential opportunity for combination treatments, and to anticipate when resistance may arise as a direct result of the immune cells and Siglecs present within the tumour.

Radiotherapy (RT) can also have a significant effect on the cell glycome, affecting which sialoglycans are present within tumours following this standard-of-care treatment. The cancer type and form of RT given can have varying effects on the expression of sialoglycans and Siglecs in the TME, which can be either beneficial or detrimental to a patient. While RT would ideally only target cancer cells, the whole tumour is routinely exposed to radiation, including the immune cells within. RT induces extreme stress on cells, particularly on the endoplasmic reticulum, and mostly achieves its cytotoxic effect on cells by widely inducing DNA damage. This cellular stress can increase signalling via pathways involving ATM, NF-κB, and IRF, which can subsequently up-regulate the expression of some sialytransferases [90–92].

The expression of several sialyltransferases is directly increased following radiation. In colon cancer cells, ionising radiation increased ST6Gal1 expression, which led to an increase in sialyation of the integrin β1 protein [93], while expression of ST3Gal5 was significantly increased in the liver of mice following whole-body radiation, which correlated with an increase in α2-3 sialyation within the liver [94]. The sialyltransferase ST3Gal4 is shown to be up-regulated in triple-negative breast cancer patients following RT, which was hypothesised to confer resistance by sialylating HSP90B1 [95]. The sialylated form of this protein is trafficked to the ER, where it effectively clears radiation-induced misfolded proteins, preventing the accumulation of reactive oxygen species and reducing the ER stress within the cell. This protects these cells from radiation-induced cell death and confers a selective advantage to those cells expressing elevated ST3Gal4. While few studies have investigated the expression of specific sialoglycans following radiation, it is likely that other proteins are also hypersialylated similarly, owing to increased expression of sialyltransferases, and that this may similarly lead to RT resistance by reducing cellular stress.

Additional evidence suggests that radiation-induced changes in sialylation are not limited to individual enzymes, but may reflect a broader remodelling of glycosylation pathways that promotes tumour survival following therapy. Ionising radiation has been shown to alter the expression of multiple glycosyltransferases and glycan-modifying enzymes, leading to changes in cell-surface sialoglycan composition that influence signalling, adhesion, and immune recognition [96]. In particular, radiation-induced glycomic reprogramming has been associated with increased levels of terminal sialylation, which can enhance activation of pro-survival pathways and reduce susceptibility to stress-induced apoptosis. Consistent with this, recent studies have reported that altered sialylation following RT contributes to the development of a more immunosuppressive tumour microenvironment, partly through increased engagement of inhibitory Siglec receptors on immune cells, thereby reducing anti-tumour immune responses [97]. In addition, transcriptomic and glycoproteomic analyses of irradiated tumour models have identified coordinated up-regulation of multiple sialyltransferases and related glycosylation enzymes, supporting the idea that radiation can drive a global shift towards a hypersialylated phenotype that promotes cellular adaptation to oxidative and endoplasmic reticulum stress [98]. Together, these findings suggest that enhanced sialylation represents a generalised stress-adaptation mechanism following radiation exposure, and that targeting sialyltransferase activity or sialoglycan–Siglec interactions may provide a strategy to overcome RT resistance.

While it has been demonstrated that routine cancer treatments can influence the level of sialoglycans expressed on cancer cells, it is likely that cancer cells adapt to this. It is reported that a loss or down-regulation of one form of sialoglycan can result in a compensatory mechanism whereby the expression of other sialoglycans is up-regulated to compensate for the loss. In breast cancer cells with ST6Gal1 knockout, an up-regulation in α2-3-linked sialoglycans is observed [99]. An increase in the expression of one type of sialoglycan may effectively mask the effect of losing the other.

## Discussion

**Figure 4:**
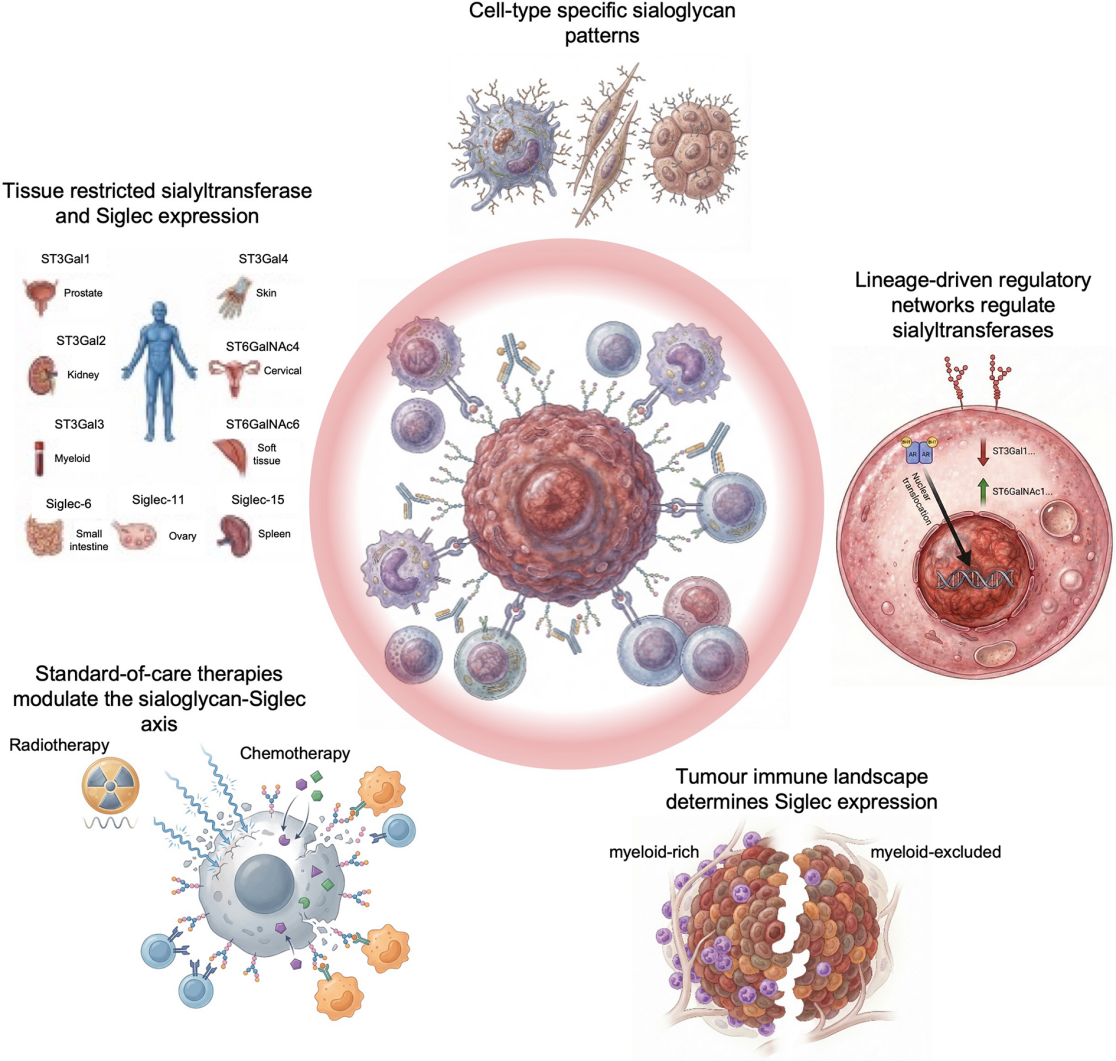
Considerations for the translation of sialoglycan-Siglec targeted cancer therapies. Key considerations for the translation of sialoglycan–Siglec biology towards effective therapeutic strategies to treat specific cancer types.

A key theme emerging from recent work is that the sialoglycan–Siglec axis is not a uniform ‘pan-cancer’ pathway, but a context-dependent system shaped by tissue identity, lineage state, and the evolving immune microenvironment (Figure 4). Basal, developmentally encoded expression of sialyltransferase families constrains which linkages and scaffolds a given tissue can generate, meaning that tumours often amplify or redistribute pre-existing sialylation capacities rather than adopting entirely new glycosylation programmes. This helps explain why different cancers display distinct sialoglycan repertoires and why the same sialyltransferase can have divergent functional consequences depending on glycan acceptor availability and cellular composition within the tumour.

While tissue-of-origin programmes help define basal glycosylation capacity, tumour-associated hypersialylation cannot always be explained by lineage alone. In several cancer types, sialyltransferase expression and sialoglycan abundance exceed that observed in the corresponding normal tissues, indicating that oncogenic signalling, epigenetic dysregulation, and stromal interactions may drive glycosylation programmes beyond their physiological baseline. These observations highlight that tissue context constrains but does not strictly determine the sialoglycan landscape in cancer.

Single-cell and functional studies increasingly implicate the tumour stroma as an active driver of immunosuppressive sialylation. Cancer-associated fibroblasts can become hypersialylated and suppress T-cell and myeloid activity via Siglec engagement, positioning stromal glycosylation as a contributor to immune exclusion and therapy resistance. This stromal dimension also emphasises that ‘tumour sialylation’ reflects the combined output of malignant cells and non-malignant compartments, each governed by distinct regulatory inputs. Dissecting which cell types produce which Siglec ligands, and how this varies across CAF subsets, macrophage states, and tumour architectures, will be essential for designing interventions with meaningful selectivity.

Mechanistically, transcription factors and lineage-determining regulators provide a direct link between cell identity programmes and glycosylation. Examples including SOX2 and MYC illustrate that oncogenic and progenitor-like states can transcriptionally activate specific sialyltransferases, yielding surface glycan changes that promote immune evasion and metastatic traits (e.g., Siglec ligands and sLeX). In prostate cancer, AR signalling adds another layer of control, where hormonal and oncogenic networks can converge on sialyltransferase regulation and shape ligand availability for inhibitory Siglecs. These findings suggest that glycosylation is embedded within broader transcriptional circuits, and that therapeutic perturbation of signalling pathways may have unintended glyco-immune consequences.

Standard therapies further complicate this landscape by dynamically altering both ligand expression and the immune context in which Siglecs operate. Hormone blockade, kinase inhibition, chemotherapy, and RT can shift sialyltransferase expression, remodel the glycocalyx, and change immune infiltration and polarisation, thereby reconfiguring which Siglecs are present and whether sialoglycans function predominantly as immune checkpoints, adhesion ligands, or barriers to drug penetration. This raises two implications for translation: firstly, sialoglycan–Siglec targeting may be most effective when aligned with tumour context (tissue origin, lineage state, and immune composition) and treatment stage; secondly, rational combination strategies will likely be required to prevent compensatory changes, including substitution between α2-3- and α2-6-linked sialylation or up-regulation of alternative immune checkpoints.

Overall, the field is moving towards a view of tumour sialylation as an ecosystem governed by tissue-intrinsic glycosylation programmes and therapy-responsive regulatory networks. Future priorities include standardised methods to map functional Siglec ligands *in situ*, causal dissection of cell-type-specific sialyltransferase programmes, and identification of biomarkers that predict dependence on the sialoglycan–Siglec axis. These advances should support more precise therapeutic targeting of sialylation and Siglec signalling, with the goal of relieving immune suppression without disrupting essential homeostatic glycosylation in healthy tissues.

## Open Access

Open access for this article was enabled by the participation of Newcastle University in an all-inclusive Read & Publish agreement with Portland Press and the Biochemical Society under an agreement with JISC.

## Abbreviations

AR: androgen receptor
CAFs: cancer-associated fibroblasts
MDSCs: myeloid-derived suppressor cells
NSCLC: non-small cell lung cancer
RT: radiotherapy
Siglecs: sialic acid-binding immunoglobulin-like lectins
sLeX: sialyl Lewis X
TME: tumour microenvironment
